# Media Generation and Pharmacy Regulatory Authority Awareness

**DOI:** 10.1177/87551225211051593

**Published:** 2022-01-10

**Authors:** Todd A. Boyle, Bobbi Morrison, Thomas Mahaffey

**Affiliations:** 1 St. Francis Xavier University, Antigonish, Nova Scotia, Canada

**Keywords:** pharmacy regulatory authority, public awareness, institutional trust, community pharmacy, regulation

## Abstract

**Background:** Professional regulatory authorities play a critical role in protecting public interest. Yet, there is a growing view that trust in regulatory authorities may be on the decline. **Objective:** Awareness has been identified as important for maintaining trust. However, research that examines public awareness and trust in pharmacy regulatory authorities (PRAs) is lacking. This research explores public awareness and trust of PRAs and presents recommendations to enhance PRA communication strategies. **Methods:** An online survey was conducted with the Nova Scotia (Canada) public in 2020. Adopting classifications from the Communications literature, 3 media generations were explored: newspaper, television, and the Internet. The χ^2^ test of independence and Kruskal-Wallis *H* test were adopted to explore differences between the generations. **Results:** Six hundred sixty-two usable surveys were obtained. Over 80% of those surveyed were aware of the existence of the PRA. Those who had heard of the PRA were most aware of its operational responsibilities and less aware of its governance. The Internet Generation was more aware that the PRA includes members of the public in its decision making than expected and showed increased trust toward the PRA versus the other media generations. **Conclusion:** The findings should help inform PRA communication plans and set baselines to assess whether such plans enhance awareness. Future studies should explore additional aspects of PRA awareness and trust, perform comparisons across pharmacy jurisdictions, and develop and test models of the relationship between PRA awareness and various dimensions of institutional trust.

## Introduction

Professional regulatory authorities play a critical role in protecting public interest. Broad expectations of regulatory authorities, regardless of profession, include establishing agreed upon entry to practice standards, ensuring continued competency and practice standards, enforcing the standards, and dealing with violations.^1^ Within a pharmacy context, pharmacy regulatory authorities (PRAs)

have the authority and responsibility to establish performance, technical, ethical and educational criteria to guide their profession, and to license individuals and their practice environments. They have a mandate of public protection and they protect the public by ensuring that the established licensure and performance requirements are met or exceeded and for disciplinary actions when the standards are not met.^2^

While differences may exist across jurisdictions, key responsibilities of PRAs may include registering and licensing pharmacists, pharmacy technicians, and community pharmacies; developing quality assurance guidelines within pharmacy practice; developing and implementing legislation and practice standards; and ensuring professional accountability.^3^

Despite a focus on protecting the public, there is a growing view that trust in regulatory authorities may be on the decline.^4,5^ Yet, within a pharmacy context, the need for high levels of public trust in PRAs is critical given the frequent contact between the public and PRA registrants (ie, pharmacists, pharmacy technicians) and the changing scope of pharmacy practice. It is therefore important for PRAs to know to what extent they are trusted by the public and assess if efforts are needed by the PRA to enhance levels of trust. Awareness is important for establishing and maintaining such trust.^6^ However, while public awareness has been identified as an important consideration of self-regulation,^7-10^ research indicates a general lack of public awareness of regulation within health professions. For example, Yam et al^7^ found low public awareness of regulation of medical professionals in Hong Kong and highlighted that “there is a significant gap between public expectations and understanding of the existing medical regulation and the actual policies and practices.” Within a pharmacy context, Gregory and Austin^11^ found low public awareness that Canadian pharmacists are regulated by provincial PRAs.

Understanding how much the public knows, and does not know, about the key PRA roles and responsibilities is important. Recommendations are emerging for health profession regulators to develop communication plans to increase public awareness, enhance public engagement, and illustrate relevancy in order to build or maintain trust.^8,12^ Understanding existing awareness levels will help inform such communication plans (eg, key messages, communication mediums) and establish baselines to assess whether such plans are effectively enhancing public awareness. In addition, increased awareness of PRA activities may also enhance trustworthiness of the pharmacy profession in general. For example, despite detecting low public awareness that Canadian pharmacists are regulated by provincial PRAs, Gregory and Austin^11^ also revealed that respondents viewed the information positively on becoming aware, suggesting public awareness as a potential means of enhancing the trustworthiness of the pharmacy profession.

However, despite the need to better understand public awareness of PRAs, empirical research exploring awareness of the specific roles, responsibilities, and governance of PRAs is lacking. There are a number of ways that the public may become aware of the roles and responsibilities of the PRA. Likewise, PRA initiatives aimed at building public trust may use several communication mediums. Examples may include PRA references in newspapers and other print media, radio and television (TV) interviews with PRA executives, and the PRA’s social media presence and webpage.

Care must be taken in selecting such mediums, as focusing on only one (eg, social media) or a few (eg, newspapers, brochures) outlets runs the risk of missing large groups that do not have a strong attachment to such media. Van der Goot et al^13^ highlight that communication scholars have suggested the presence of media generations, where “generations that are young when a particular medium becomes popular may have a stronger attachment to that medium than do previous or later generations.”^(p291)^ The oldest media generation, the Newspaper Generation, grew up with newspapers as the primary communication media. Communication scholars have identified that this generation are the most frequent readers today. The TV Generation grew up with the rise of TV and are less inclined to read versus the Newspaper Generation. The Internet (Net) Generation grew up with information technology and the internet.^13^ This generation grew up with accesses to far greater and instantaneous content versus previous generations. While a PRA may undertake efforts to enhance awareness and trust, the success of such efforts may be affected by media generations. Therefore, in addition to exploring overall levels of PRA awareness and trust, this study explores how such awareness and trust may differ based on media generation. The objectives of the study were to

- Identify current levels of public awareness and trust of PRAs
- Determine how awareness and trust differs based on media generation
- Present recommendations to PRAs to enhance awareness and trust

## Methods

### Measures

To explore public awareness of PRAs, this research focused on the pharmacy jurisdiction located in the Canadian province of Nova Scotia. This PRA’s “legislated mandate is to maintain standards of practice and professional accountability in the practice of pharmacy, thereby supporting optimal patient care.”^14^ Key responsibilities of this PRA includes registration and licensing, quality assurance, legislation and practice standards, and professional accountability.^14^ The powers of the PRA are exercised by a council that is composed of 8 registrants elected by registrants, 3 public representatives that are appointed, and the director of the only university pharmacy program in the province. In Nova Scotia, the regulation of pharmacists uses a model of statutory self-regulation.^15^ This is not always the case in other jurisdictions.

To capture overall awareness of the PRA, 8 statements relating to the key roles, responsibilities, and governance of the PRA were developed in consultation with the Nova Scotia PRA. These statements included the following: (1) I was aware that the PRA is the only organization in the province that can license pharmacists to practice; (2) I was aware that the PRA is the only organization in the province that can license pharmacy technicians to practice; (3) I was aware that the PRA, and not the government, establishes the rules for the day-to-day practice of pharmacy; (4) I was aware that the PRA has the responsibility of ensuring quality care is provided by pharmacy professionals; (5) I was aware that complaints about pharmacists, pharmacy technicians, and community pharmacies should be directed to the PRA; (6) I was aware that complaints against pharmacists, pharmacy technicians, and community pharmacies are investigated by the PRA; (7) I was aware that when pharmacy professionals do not provide quality care, the PRA will take action; and (8) I was aware that the PRA includes members of the public in its decision making.

Pharmacy regulatory authority trust for this study was captured by 3 general questions that were developed in consultation with the Nova Scotia PRA and following a review of the institutional trust dimensions suggested by the Organization for Economic and Cooperative Development (OECD).^16^ For this study, trust items that focused on the PRA’s general mandate of protecting the public and an overall assessment of trust were considered. PRA trust was captured using 3 questions, specifically: (1) I can count on the PRA to keep me safe from harm in pharmacies; (2) the PRA puts the needs of the public before the needs of pharmacy professionals; and (3) all in all, I have complete trust in the PRA. This research classified the Newspaper Generation as those aged 65+ years, TV Generation as those aged 45 to 64 years, and the Net Generation as 25 to 44 years of age, closely approximating the age groups presented by van der Goot et al.^13^

### Instrument

This research formed part of a larger study that explored public attitudes toward various aspects of community pharmacy practice. To explore such attitudes, an online survey questionnaire was developed and administered to the Nova Scotia public. The 8 PRA awareness questions were presented to the public, with the PRA’s formal name used in the survey, and captured as a Yes/No dichotomy. The PRA trust questions, with the PRA’s formal name also used, were captured with a 5-point Likert-type scale with Strongly Disagree (1) and Strongly Agree (5) as anchors. Individuals were also asked various screening (ie, have you heard of the PRA, do you currently work in a pharmacy) questions.

The online questionnaire was prepared and administered using Qualtrics software and presented in English. Quota sampling was applied to achieve representativeness by age, household income, locale, and gender. Dynata, a third-party survey sampling company, was selected to recruit respondents and present participants with a link to the online Qualtrics survey. Prior to the full launch, the survey instrument was pretested with 35 respondents for content validity and to assess comprehension and potential issues with the technology. A soft launch with 100 respondents followed. Detecting no issues with soft launch responses, those who completed the survey were retained in the final data set. No personally identifiable information on the respondents was collected by the researchers. Prior to analysis, the data were cleaned to remove responses from speeders (those with a completion time of <6 minutes), those who did not complete the survey, and those under the age of 25 years (ie, outside of the 3 generations under study). The St. Francis Xavier University Research Ethics Board reviewed and provided ethics approval for this project.

### Statistical Approach

The χ^2^ test of independence was adopted to explore the association between awareness of the PRA and media generation. Given that PRA awareness consisted of 8 items, and therefore multiple hypothesis tests, the Bonferroni correction was applied to better control for Type I error. As the χ^2^ test of independence is an omnibus test, a post hoc analysis was conducted, through examination of the adjusted standardized residuals with a Bonferroni correction applied, in cases of statistically significant findings. The Kruskal-Wallis *H* test was used to explore differences in PRA trust based on media generations. In the case of a statistically significant finding, post hoc follow-up testing using Dunn’s test with Bonferroni-adjusted *P* values were used to explore where perceptions differed. All statistical analyses were performed using IBM SPSS Statistics Version 26.

## Results

### Data Collection

The online survey was conducted in May and June 2020 and occurred in Nova Scotia, Canada. An initial sample of 676 surveys spanning the 3 media generations were collected. Given that various members of the pharmacy team are regulated by the PRA, it was expected that those who work in a pharmacy would have increased awareness of the PRA versus members of the general public. As such, the first screening question captured whether the respondent worked in a pharmacy. Of the 676 initial respondents, 12 identified as working in a pharmacy, while 2 did not answer the question. These 12 respondents were removed from further analysis, as were the 2 respondents who did not answer the question as it could not be confirmed that they did not work in a pharmacy. This reduced the usable sample size to 662.

The second screening question asked respondents whether they had ever heard of the official name of the PRA. Of the 662 respondents, 532 (80.4%) had heard of the provincial PRA, 102 (15.4%) had not, and 28 (4.2%) chose not to answer the question. The χ^2^ test of independence indicated no differences in familiarity of the official name of the PRA based on media generation (*P* = .988). In order to capture awareness of the PRA beyond name recognition, only respondents who had at least heard of the formal name of the PRA were included in further analysis. As a result, the final usable sample size for this study was reduced to 532, which resulted in a margin of error of 4.19% at 95% confidence interval.^17^

### Demographics

The sample composed 150 (28.2%) respondents from the Newspaper Generation, 221 (41.5%) respondents from the TV Generation, and 161 (30.3%) respondents from the Net Generation. In addition, the sample consisted of 255 (47.9%) respondents that identified as female, 274 (51.5%) respondents that identified as male, and 1 (0.2%) respondent that identified as nonbinary. Two (0.4%) respondents chose not to answer the question about gender. Respondents were from a mix of urban (198 respondents, 37.2%), suburban (157 respondents, 29.5%), and rural (172 respondents, 32.3%) locations. Five respondents (0.9%) chose not to state their location. In terms of education, 74 (13.9%) had an education of high school or less, 97 (18.2%) had some college or university training, 271 (50.9%) had completed college/university, 87 (16.4%) had a postgraduate degree, and 3 (0.6%) chose not to answer the question. In terms of perceived health status, 71 (13.3%) perceived their health to be not very good or worse, 238 (44.7%) viewed their health as average, and 222 (41.7%) viewed their heath as very good or better. One respondent (0.2%) chose not to state their perceived health status.

### Awareness and Trust

Eight items were presented to respondents to explore PRA awareness. Table 1 presents the public’s awareness of various responsibilities and characteristics of the PRA. Results indicated that a majority of respondents were aware of all PRA responsibilities and characteristics included in the survey except the statement that members of the public are included in PRA decision making. A substantial proportion of respondents reported an awareness about the PRA’s responsibility in ensuring quality care is provided by pharmacy professionals and its role in licensing pharmacists.

**Table 1.** Public Awareness of the PRA.

	Not aware, n (%)	Aware, n (%)	Total N
I was aware that . . .			
[the PRA*] is the only organization in the province that could license pharmacists to practice.	126 (25.8%)	362 (74.2%)	488
[the PRA*] is the only organization in the province that could license pharmacy technicians to practice.	198 (41.1%)	284 (58.9%)	482
[the PRA*], and not the government, establishes the rules for the day-to-day practice of pharmacy.	206 (44.1%)	261 (55.8%)	467
[the PRA*] has the responsibility of ensuring quality care is provided by pharmacy professionals.	118 (23.8%)	377 (76.2%)	495
Complaints about pharmacists, pharmacy technicians, and community pharmacies should be directed to [the PRA*].	155 (32.2%)	326 (67.8%)	481
Complaints against pharmacists, pharmacy technicians, and community pharmacies are investigated by [the PRA*].	137 (29.3%)	330 (70.7%)	467
When pharmacy professionals do not provide quality care, [the PRA*] will take action.	157 (34.5%)	298 (65.5%)	455
[the PRA*] includes members of the public in its decision making.	316 (70.7%)	131 (29.3%)	447

Abbreviation: PRA, pharmacy regulatory authority.

* The official name of the PRA was used in the survey.

Results of the χ^2^ test of independence indicated statistically significant associations between media generation and one aspect of PRA awareness. Specifically, a statistically significant association was found between media generation and awareness that the PRA includes members of the public in its decision making (*P* = .000). Follow-up analysis using the cells’ adjusted residuals with a Bonferroni correction found greater awareness of this characteristic among the Net Generation than was expected (observed = 66, expected = 43, adjusted residual = 5.1, *P* = .000).

Three general questions were used to capture PRA trust. Results indicated that the public places moderate to high levels of trust in the PRA. The combined data showed high levels of trust that the PRA keeps the respondent safe from harm in pharmacies (median = 4). Similarly, levels of complete trust in the PRA (median = 4) were also high. Respondents were neutral in their assessment that the PRA puts the needs of the public before the needs of pharmacy professionals (median = 3). The Kruskal-Wallis *H* test was used to assess how such levels of trust in PRAs may differ based on media generation. Results of this test indicated statistically significant differences between the 3 generations in their views that the PRA puts the needs of the public before the needs of pharmacy professionals (*P* = .000). Pairwise comparisons using Dunn’s test with Bonferroni-adjusted *P* values indicated that Net Generation scores were higher (median = 4, mean rank = 248.7) than those of the TV (median = 3, mean rank = 201.9, *P* = .001) and Newspaper (median = 3, mean rank = 178.9, *P* = .000) generations. A statistically significant difference was also found in the extent to which the respondent has complete trust in the PRA (*P* = .000). Net Generation scores were higher (median = 4, mean rank = 279.6) than those of the TV (median = 4, mean rank = 244.8, *P* = .040) and Newspaper (median = 3, mean rank = 197.4, *P* = .000) generations. TV generation scores were also statistically shown to be higher than the Newspaper generation (*P* = .003). No statistically significant differences were found between media generations and assessments that the PRA keeps the respondent safe from harm in pharmacies (*P* = .060).

## Discussion

Pharmacy regulatory authorities play a critical role in the safe delivery of health care through activities such as registering and licensing pharmacists, pharmacy technicians, and community pharmacies, developing quality assurance guidelines and practice standards, and providing professional accountability.^2^ Given broader challenges of trust in regulatory bodies in general,^4,5^ PRAs should be mindful of the extent that the public trusts the PRA to carry out such activities and explore how to build or sustain high levels of public trust. As shown in other organizational contexts, public awareness is important for building and maintaining organizational trust^18,19^ and communication strategies used by health organizations have been credited with increased awareness of the organization.^20^ Therefore, an initial step in enhancing trust in the PRA is to ensure adequate public awareness of the PRA which may be accomplished by preparing and executing a communication plan with this intent.

- *Recommendation 1*: PRAs should develop a communication plan for the purpose of enhancing trust through awareness.

Overall, the findings highlight basic awareness of the subject PRA and its key roles and responsibilities. Over 80% of those surveyed were aware of the existence of the subject PRA, albeit not aware of all of its key roles and responsibilities. Those who had heard of the subject PRA were most aware of its operational responsibilities, specifically ensuring that pharmacy professionals provide quality care, licensing pharmacists, and investigating complaints. The public appeared to be less aware of the governance of the PRA, with the public the least aware that the PRA includes members of the public in its decision making, and that the PRA, and not the government, establishes the rules for the day-to-day practice of pharmacy. As highlighted by Yam et al,^7^ “there is emerging emphasis on increasing involvement of lay people in Medical Councils for greater transparency and accountability.”^(p94)^ And while public representation is common in PRA decision making, the public remains somewhat unaware of such involvement, despite its value in enhancing transparency and accountability. As a result, for PRAs that include public representation in decision making (eg, boards, investigation committees), building awareness of such should form part of PRAs’ communication plans. Overall, the findings indicate the need for communication strategies to not only focus on the specific roles of the PRA, such as licensing and investigations, but also on how key decisions are made and the inclusion of public representation in such decisions.

- *Recommendation 2*: PRA communication strategies should not only focus on enhancing awareness of the roles and responsibilities of the PRA, but also its governance with an emphasis on public participation in decision making.

The results revealed differences in PRA awareness based on media generation with the Net Generation more aware than expected that the PRA includes members of the public in decision making. The findings speak to the need for a communication strategy that is multifaceted and includes various modes of communication. Public health communication campaigns utilizing mass media have proven effective at influencing knowledge and attitudes, and while most campaigns have used television, there has been an evolution to digital media.^21^ In the context of health-related professions, regulators are recommended to make greater use of social media in order to remain relevant, facilitate communication, increase public awareness, and enhance engagement.^8^ Similar to recommendations for public sector organizations, PRAs could utilize social media to enhance their media relations to accelerate dissemination of news content and heighten organizational visibility and legitimacy among the public.^22^

However, the findings indicate that increased posting and expanded use (ie, multiple platforms) of social media may not be effective in enhancing awareness and trust among all members of the public, especially those from the TV and Newspaper generations. Media use frequencies have been shown to differ among generations^13^ and are pronounced by the digital divide.^23^ Therefore, in addition to social media, print (eg, newspapers, in-pharmacy brochures), radio, and other communication media are also needed to enhance public awareness of the PRA. Additionally, where public education videos have proven effective in increasing awareness about the pharmacy profession in experimental settings,^24^ video messaging, whether delivered via social media and alternative channels, should be incorporated into the communication strategy.

- *Recommendation 3*: PRA communication strategies should utilize multiple communication media, including an active social media presence, traditional mediums (eg, TV, radio, print) and incorporate video content where such media permit.

Overall, the public places high levels of overall trust in the PRA, as well as trust in keeping individuals safe from harm in pharmacies. However, trust that the PRA puts the needs of the public before the needs of pharmacy professionals scored lower. The Net Generation appeared to be more trusting of the PRA in general and scored this latter trust item higher than the other 2 generations. The findings suggest that especially for the older media generations, there is a need for PRAs to clearly differentiate themselves from professional pharmacist associations, who are responsible for protecting the interests of practicing pharmacists. This may include the PRA, as part of its communication strategy, outlining the key roles and responsibilities of professional pharmacists’ associations, and how they also play a key role in protecting the public with regard to pharmacy practice.

- *Recommendation 4*: PRA communication strategies should include messaging that clearly differentiates between the roles and responsibilities of PRAs and professional pharmacists’ associations, while highlighting how professional pharmacists’ associations also protect the public with regard to pharmacy practice.

Finally, public trust in the PRA should not be viewed as static and may change given the dynamic nature of pharmacy practice (eg, expanded pharmacy services), fallout from high-profile medication incidents, or access to pharmaceuticals, among others. The public should be aware of the PRA’s roles and responsibilities with regard to such issues, as an early step to help maintain or enhance PRA trust. As such, PRAs should first establish baseline levels of public awareness, set initial benchmarks for increasing awareness, periodically assess whether such benchmarks are being met, and assess public awareness of the PRA’s roles and responsibilities in higher profile issues.

- *Recommendation 5*: PRAs should establish baseline levels of public awareness, set initial benchmarks for increasing awareness, and periodically assess to determine if such benchmarks are being met. In addition to general benchmarks, PRAs should explore public awareness of the PRA’s roles and responsibilities in more specific and higher profile issues such as expanded pharmacy services, medication incidents, and drug supply shortages.

There are a number of limitations to this study. This research collected data using an online survey. The use of the online survey may have skewed the results to those that are more internet savvy. The trust items selected for this research related to protecting the public and an overall assessment. Additional trust items developed using dimensions suggested by the OECD^16^ should be considered in future studies. This research focused on a single pharmacy jurisdiction and on individuals who already had some basic awareness of the PRA by recognizing its formal name. While this research, therefore, excluded respondents who did not recognize the PRA, an exploration of this segment is recommended for future research. While this study focused on a single factor that affects PRA awareness (ie, media generation), future studies should explore additional variables such as gender identity, location, or cultural identity. Beyond public awareness, future research should also consider the awareness that pharmacy staff and other health care professionals (eg, physicians) have of the roles and responsibilities of PRAs, especially as many stakeholders may not understand the distinctions between regulators and advocacy bodies.

Despite these limitations, this research has important implications to both PRAs and researchers. This study provides PRAs with issues to consider when they develop their own public communication strategy and provides a means to develop baseline measures of public awareness. Public awareness of PRAs is very much an underexplored area of research. As such, this research contributes to the body of knowledge on PRA awareness by gauging aspects of the PRA that the public is more (or less) likely to be aware of and exploring how such awareness may differ based on media generation.

## Conclusion

The role of the PRA in ensuring safe delivery of pharmacy services is not well understood among the public. Using the case of a single pharmacy jurisdiction, this research explores public awareness of the key roles and responsibilities of PRAs and overall levels of public trust in PRAs. The findings highlight basic awareness of the PRA. Those who have heard of the PRA were most aware of its operational responsibilities but less aware of its governance. Respondents placed moderate to high levels of trust in the PRA, with the Net Generation being more trusting of the PRA in general. Recommendations such as blending online and more traditional communication mediums, increasing details of PRA governance, and outlining how PRAs differ from professional pharmacists’ associations should be considered by PRAs when developing their public communications strategy.
